# Individual, Community, and Health Facility Predictors of Postnatal Care Utilization in Rural Tanzania: A Multilevel Analysis

**DOI:** 10.9745/GHSP-D-22-00502

**Published:** 2023-08-28

**Authors:** Florina Serbanescu, Purni Abeysekara, Alicia Ruiz, Michelle Schmitz, Sunday Dominico, Jason Hsia, Paul Stupp

**Affiliations:** a Division of Reproductive Health, U.S. Centers for Disease Control and Prevention, Atlanta, GA, USA.; b Thamini Uhai, Dar es Salaam, Tanzania.; c Division of Population Health, U.S. Centers for Disease Control and Prevention, Atlanta, GA, USA.; d Division of Global HIV and TB, U.S. Centers for Disease Control and Prevention, Atlanta, GA, USA.

## Abstract

**Introduction:**

Postnatal care (PNC) is an underused service in the continuum of care for mothers and infants in sub-Saharan Africa. There is little evidence on health facility characteristics that influence PNC utilization. Understanding PNC use in the context of individual, community, and health facility characteristics may help in the development of programs for increased use.

**Methods:**

We analyzed data from 4,353 women with recent births in Kigoma Region, Tanzania, and their use of PNC (defined as at least 1 checkup in a health facility in the region within 42 days of delivery). We used a mixed-effects multilevel logistic regression analysis to explain PNC use while accounting for household, individual, and community characteristics from a regionwide population-based reproductive health survey and for distance to and adequacy of proximal health facilities from a health facility assessment.

**Results:**

PNC utilization rate was low (15.9%). Women had significantly greater odds of PNC if they had a high level of decision-making autonomy (adjusted odds ratio [aOR]: 1.56; 95% confidence interval [CI]=1.11, 2.17); had a companion at birth (aOR: 1.57; 95% CI=1.19, 2.07); had cesarean delivery (aOR: 2.27; 95% CI=1.47, 3.48); resided in Kasulu district (aOR: 3.28; 95% CI=1.94, 5.52); or resided in a community that had at least 1 adequate health facility within 5 km (aOR: 2.15; 95% CI=1.06, 3.88).

**Conclusion:**

Women’s decision-making autonomy and presence of companionship at birth, as well as proximity to a health facility with adequate infrastructure, equipment, and workforce, were associated with increased PNC use. More efforts toward advocating for the health benefits of PNC using multiple channels and increasing quality of care in health facilities, including companionship at birth, can increase utilization rates.

## Abstract (precis)

PNC visits are an opportunity to promote physical and emotional recovery, offer infant care and feeding advice, detect and treat postpartum complications, and discuss birth spacing. Offering PNC in nearby health facilities with adequate quality of care can increase usage rates.

## Teaser

**Key Findings:**

- Coverage for postnatal care (PNC) in a remote, mostly rural region of Tanzania was low, with 5 of 6 women not using facility-based postnatal checkups.
- Controlling for all other factors, we found that several individual and community characteristics were associated with PNC, including decision-making autonomy, presence of a birth companion for support during labor and delivery, cesarean delivery, district of residence, and presence of an adequate health facility within 5 km travel distance.
- Increasing the use of PNC services would require improving the adequacy of care in proximal health facilities, empowering women to use preventive services, and increasing women’s and communities’ birth preparedness.

**Key Implications:**

- Efforts to increase quality of care in lower-level facilities to ensure high-quality services will close the “quality gap” and improve PNC coverage.
- More research is needed to understand and address barriers to using facility PNC.

## INTRODUCTION

Roughly 303,000 women died in 2015 worldwide from complications during and after pregnancy, with most of these deaths occurring in low-resource settings.^1^ In 2017, there were approximately 5.4 million deaths of children aged 5 years and younger, with 2.5 million of these deaths happening within the first month of birth.^2^ High levels of maternal (556 per 100,000 live births) and neonatal (25 per 1,000 live births) mortality persist in Tanzania; ^3^ these rates are significantly higher than United Nations Sustainable Development Goals 3.1 and 3.2, which aim to reduce maternal mortality to 70 per 100,000 live births and neonatal mortality to 12 per 1,000 live births globally.^4^

Although quality maternity care services during pregnancy, labor, and delivery are critical to averting many maternal and newborn deaths, the continuum of care during the “fourth trimester” is equally important.^5^ Integrated postnatal care (PNC) for mothers and neonates includes the prevention, early detection, and treatment of complications; support for breastfeeding; nutrition and hygiene; counseling on birth spacing and family planning; and provision of immunization and HIV services. In Tanzania, the Ministry of Health, Community Development, Gender, Elderly and Children (MOHCDGEC) promotes this PNC content and recommends that mothers and infants receive a minimum of 4 PNC checkups during the first 6 weeks post-delivery: within 48 hours and between 3–7 days, 8–28 days, and 29–42 days after delivery.^6^ However, national population-based estimates show that only 34% of women who gave birth in 2013–2016 received PNC within 2 days of delivery and only another 2% within 3–41 days from birth; these rates did not change substantially over time.^3^ Nurse-midwives and clinicians in public and private health facilities are the main providers of PNC services.^3^ While the national guidelines promote community mobilization and involvement, the emphasis is on improving community and individual awareness about receiving PNC in health facilities.^6^

The continuum of care during the “fourth trimester” is equally important as care during pregnancy, labor, and delivery to avert many maternal and newborn deaths.

More than one-half of pregnancy-related deaths globally occur after birth.^7^ Postnatal visits are essential in detecting and treating postpartum complications, such as postpartum depression, and in promoting infant care and feeding, birth spacing, and physical recovery.^6^ Despite wide recognition of its benefits, PNC is underused globally, particularly where maternal mortality is the highest.^1^^,^^8^

Several studies have recognized individual characteristics that are positively associated with PNC utilization. Woman’s education and literacy, partner’s education, household wealth, parity, type of delivery facility, and antenatal care (ANC) use were related to PNC utilization in some but not all studies in sub-Saharan Africa.^8^^–^^14^ Interpersonal factors, such as women’s decision-making autonomy, may also be associated with PNC use.^15^ Studies specific to Tanzania found that cesarean delivery and high community use of family planning increased facility PNC.^16^^,^^17^ Shortages of staff, equipment, and supplies were common reasons for not seeking PNC at health facilities.^18^

Although results varied, several studies identified proximity to a health facility as a predictor of PNC use, using either Euclidean straight-line distances or modeled travel time.^10^^,^^16^^,^^17^^,^^19^^,^^20^ Due to the complexities of measuring quality, fewer studies examined the influence of the quality of maternal care in health facilities. Although studies found that high quality of care is associated with delivering in a facility or accessing ANC,^21^^–^^27^ no study examined its influence on PNC use. These findings suggest that a positive association may exist between quality of maternity care and PNC utilization.

Most studies assessing whether the health service environment, including geographic access and quality of care, is associated with care-seeking behaviors usually use ecological linkages, where information on health care-seeking behaviors from population survey data in a region is linked to health service provision data aggregated within the same geographical areas.^28^ Studies that directly link respondents with the characteristics of health facilities they used for preventive maternal care services are rare.^27^

Several studies in low- and middle-income countries focused on determinants of PNC at the population level (individual and community factors),^10^^–^^12^^,^^16^^–^^20^ but there is a need to examine the association between PNC and individual and community factors and readiness of health facilities. In this analysis, we explored how a woman’s demographic characteristics, behavioral and obstetric factors, and the characteristics of her community, community proximity, and the health facility where she gave birth were associated with PNC utilization in Kigoma Region, Tanzania. This study directly links women from a large population-based survey of maternal, child, and reproductive health with the health facility environment documented through a census of health facilities providing maternity care to examine multilevel effects on using PNC services.

There is a need to examine the association between PNC and individual and community factors and readiness of health facilities.

## METHODS

### Study Setting

Kigoma Region, situated in the northwest corner of Tanzania, is part of the country’s Western Zone and borders Lake Tanganyika, the Democratic Republic of the Congo, and Burundi to the west.^29^ The largely rural region covers 45,066 square kilometers and, in 2012, had a population of 2,127,930. Population density is low, with more than 82% of the area designated as rural and the largest population residing in Kasulu district. Topographically, there is a wide range of altitudes. The lowest areas are in Kigoma rural, along Lake Tanganyika and Kigoma urban, while the highest areas (highlands) are in the northern part of the region constituted by Kibondo district. Agriculture is the main economic activity in 3 of 4 districts (Kigoma rural, Kasulu, and Kibondo), while trade and fishing are the main activities in Kigoma urban. Transportation challenges are common in all districts due to sparse population and poor roads (there was only 1 paved road in the region in 2019). Kigoma rural has the lowest access to health services due to fewer health facilities and impassable roads most of the year along Lake Tanganyika.

According to national survey estimates from 2015–2016,^3^ the Western Zone had the highest fertility rates, lowest modern contraceptive use rates, and lowest proportion of deliveries in health facilities compared to all other zones. The *National Roadmap Strategic Plan to Accelerate Reduction of Maternal, Newborn and Child Deaths in Tanzania* prioritized accelerated progress in the Lake and Western Zones (including Kigoma Region), where reproductive, maternal, and newborn health outcomes remain poorer than in other parts of the country.^30^

With the support of the Maternal and Reproductive Health in Tanzania Program (The Program), a long-term public-private partnership carried out from 2006 to 2019 in Kigoma to reduce maternal mortality, a series of facility and community interventions was implemented to strengthen emergency obstetric and neonatal care (EmONC) and improve access to and use of contraception. The Program aimed to decentralize obstetric, reproductive, and newborn care from distant hospitals to more accessible, lower-level health facilities through infrastructure upgrades, training of nonphysician health providers staffing these facilities, supportive supervision, and community outreach activities aimed to raise awareness about pregnancy-related complications, birth planning, use of preventive health services, and contraceptive methods. Strengthening service delivery at the dispensary level further expanded women’s geographic access and was coupled with strengthening the referral systems for obstetric complications.^31^ The Program was funded by Bloomberg Philanthropies and Foundation H&B Agerup and implemented by the Government of Tanzania in collaboration with Thamini Uhai and EngenderHealth. From 2012 to 2019, the Centers for Disease Control and Prevention Division of Reproductive Health (CDC) partnered with The Program to conduct evaluation activities.

### Data Sources

CDC conducted a series of monitoring and evaluation activities to assess changes in facility-based care and its utilization in Kigoma Region. This included periodic health facility assessments (HFAs), pregnancy outcome monitoring in all health facilities, and a series of 3 reproductive health surveys (RHS) that documented changes in maternal, child, and reproductive health indicators among women of reproductive age (15–49 years) regionwide. For this analysis, we used information on Kigoma women with births during January 2016–August 2018 from the 2018 Kigoma RHS. We matched women with the health facilities where they reported having received delivery care for the most recent birth and examined the adequacy of services in these facilities as documented in the 2019 HFA. We also matched communities with the proximal health facilities and assessed how proximity to a facility with adequate services in a community influences PNC use.

#### 2019 Health Facility Assessment

The HFA focused on factors that contributed to the availability and accessibility of routine and emergency obstetric care, such as adequate staffing, infrastructure, equipment and supplies, communication and referral pathways, transportation, and staff training.^32^ We collected geo-located data for the HFA during February 2019 through direct observation and verification in 197 health facilities: 6 hospitals, 27 health centers, and 164 dispensaries that provided delivery care. Collectively, these facilities provided more than 95.5% of facility delivery care in 2018 in Kigoma Region, according to the region’s health management and information system.^33^ We used all 197 facilities for assessing the community proximal facility and 145 facilities where women recently delivered for assessing adequacy of delivery facility.

We used the results of the HFA to assess facility readiness to provide routine obstetric care based on attributes considered essential for routine obstetric and newborn care across domains identified in the World Health Organization’s Service Availability and Readiness Assessment tool.^32^ We used facility readiness of care attributes across multiple domains characterizing provision of care at the delivery facility as defined in the HFA: human resources, infrastructure, processes, essential drug stock, essential equipment, facility capacity, and transportation^34^ (Table 1 and Supplement). We included additional attributes related to “general facility requirements” for routine obstetric care, as proposed by Gabrysch et al.: availability of services 24/7 and having at least 1 staff member trained in EmONC who provided delivery care.^35^ For each of these 30 attributes, a score of 1 was assigned if the item was present and a score of 0 if it was absent. There were no facilities with missing information on these 30 items; the facility scores ranged from 18 to 30. Facilities that had a score in the highest decile (i.e., having 28 or more attributes) were classified as having adequate readiness for routine maternal care services.

**TABLE 1.** Attributes of Health Facility Readiness^a^ in Routine Maternal Care

Domain	Definition^b^	Attribute
Human resources	Adequacy of staff providing delivery care (positions designated are occupied)	1. Adequate number of delivering personnel, per Ministry of Health staffing guidelines^40^ 2. At least 1 staff trained in basic emergency obstetric care^c^
Infrastructure	Basic physical features and resources at the health facility that improve and maintain the health of the mother and infant	3. Electricity available 24 hours/day and 7 days/week 4. Improved water source within 500 m of facility 5. Private room for patient consultation 6. Access to clean and functioning toilets 7. Communication equipment 8. Safe disposal of sharps and infectious waste
Essential equipment and supplies	Presence of essential and lifesaving necessary items that may improve or maintain health of the mother and infant	9. Newborn bag and mask 10. Blood pressure cuff 11. Adult stethoscope 12. Fetal stethoscope 13. Oxygen source 14. Suction apparatus (mucus extractor) 15. Sterilization equipment 16. Vacuum aspirator or D&C kit 17. Manual vacuum extractor for assisted vaginal delivery 18. Adequate delivery instrument set 19. Gloves
Essential drug stock	Supply of essential and lifesaving medicines that may improve or maintain health of the mother and infant	20. Injectable uterotonic 21. Injectable antibiotics 22. Injectable magnesium sulfate 23. Intravenous solution 24. 24. Antihypertensive medication 25. Antibiotic eye ointment for newborns
Processes	Series of tasks or methods applied at the health facility that may improve or maintain health of the mother and infant	26. Guidelines for IMPAC present in labor and delivery room 27. Partograph forms and proof of use
Facility capacity and functionality	Ability for the health facility to occupy patient beds and accomplish functions and tasks that may improve or maintain health of the mother and infant	28. Adequate number of delivery beds 29. Availability of labor and delivery services 24 hours/day and 7 days/week^c^
Transportation	Supply of transportation that is functional and available to staff that may facilitate medical care at the health facility	30. Motorized vehicle with motor fuel available for emergency referrals

Abbreviations: D&C, dilation and curettage; IMPAC, Integrated Management of Pregnancy and Childbirth.

a 2015 World Health Organization’s Service Availability and Readiness Assessment Guidelines.^34^

b To construct a facility readiness index, a score of 1 was assigned to each attribute for a maximum score of 30 attributes; a facility that met 28 or more attributes (corresponding to the highest decile of 30 attributes of the quality index) was considered to have adequate readiness of its routine maternity services.

c Having at least 1 staff member who provided delivery care trained in emergency obstetric and neonatal care and availability of services 24 hours/day and 7 days/week were added in the index construct, as proposed by other researchers.^35^

#### 2018 Kigoma Reproductive Health Survey

The 2018 RHS is a regionally representative survey exploring maternal and newborn health in Kigoma Region. The RHS used the census enumeration areas developed for the 2012 National Population and Housing Census as its sampling frame.^36^ We identified a representative sample of eligible women residents aged 15–49 years in Kigoma Region using a 2-stage cluster sample design including: (1) the selection of primary sampling units (PSUs) using a probability proportional to the size of the enumeration areas approach in the first stage, and (2) the selection of households within each selected PSU using systematic random sampling. All PSU household listings were updated during July–August 2018 to account for changes since the 2012 census.

From September to November 2018, trained interviewers visited 10,021 households and obtained informed consent before conducting household and individual interviews; the household response rate was 98.8%. Of the 10,542 eligible women identified, 10,181 (96.6%) completed interviews. At the beginning of the household interviews, household respondents gave their informed consent, and at the start of the individual interviews, eligible respondents gave their informed consent in accordance with the Tanzania requirements for human subject participation in population surveys.

No compensation of any kind was provided to respondents who agreed to voluntarily participate in the survey. Detailed descriptions of the survey methods and procedures are available elsewhere.^37^ Respondents were asked for information about their background characteristics, contraceptive behaviors and use, fertility, and lifetime pregnancy history. Women who had given birth between January 2016 and September 2018 were asked for detailed information about their most recent births, including details about ANC, delivery, and PNC, as well as the names of the facilities they went to for these services.^38^

We used information collected from the RHS to examine PNC use according to individual and community characteristics, including measures of individual and community access to adequate maternal care in facilities constructed from linkages with HFA data.

### Study Sample

From the 10,181 women who completed the RHS, we restricted our sample to 4,457 women who delivered their last baby from January 2016 to August 2018 to allow for equal exposure to PNC initiation. Of those, 787 women had used PNC, and 3,670 women had not. We excluded women who indicated that they received their first PNC later than 42 days after delivery (22 women), at a facility outside Kigoma Region (41 women), or at home (41 women). Our final analytic sample (n=4,353) comprised 683 women who used PNC at a health facility in Kigoma Region within 42 days after delivery and 3,670 women who did not use PNC.

### Measures

#### Outcome Variable

We examined PNC utilization for maternal health after last birth (live birth or stillbirth), expressed as a binary variable. Receipt of PNC was defined as having any maternal postnatal checkup at a health facility in Kigoma Region (dispensary, health center, or hospital, either government or private) at any time during the first 42 days post-delivery. PNC utilization was examined for all women with facility deliveries (“After you left the facility, did any health care provider or traditional birth attendant check on your health?”) or deliveries outside a health facility (“After the baby was born, did any health care provider or traditional birth attendant check on your health?”) who received PNC in a health facility in the region. Less than 1% of women stated they received PNC at home, and we did not include these women in our analyses of facility PNC use. While the survey separately collected information about PNC use for newborns, we decided to focus our analyses on PNC use for mothers only. First, we observed that PNC for newborn health had a substantially higher coverage but skewed toward the 4–6 weeks after delivery. Next, the Program primarily targeted PNC in the first week after delivery, when PNC use was comparable and equally low for mothers and babies. Use of PNC for newborn care spiked around 30 days after delivery, coinciding with the onset of immunization with diphtheria, tetanus, pertussis; oral polio; and hepatitis B vaccines. Lastly, coverage of PNC for the mother reflects the use by all women with recent births, including those whose babies were born dead or died soon after birth.

#### Household- and Individual-Level Characteristics

We classified household wealth status (low, middle, and high) by analyzing multiple assets present in the household, including durable goods, such as radio, television, phone, computer, refrigerator, bicycle, motorcycle, boat, animal-drawn cart, car, or truck and dwelling characteristics, such as availability of electricity, source for drinking water, toilet facilities, fuel used for cooking, main roof material, and number of rooms used for sleeping. To construct the index, each asset was assigned a relative weight generated through principal component analysis. The scores were standardized to have a standard normal distribution with a mean of zero and a standard deviation of 1, and each household was assigned a standardized score reflecting its existing set of assets and possessions.^37^^,^^38^ Individual characteristics included demographic variables, such as maternal age, literacy (defined as the ability to read a simple Swahili sentence shown on a card), work status (held a job outside the home), current marital status, parity (1, 2–4, 5 or higher), and woman’s decision-making autonomy in the household. The index was created using standard questions^39^ asking women to report which member of the household was usually responsible for 6 possible decisions: the respondent’s health care, large household purchases, household purchases for daily needs, how to use the money that the respondent brought into the household, how to use the money that her partner brought into the household, and whether the respondent was allowed to work to earn money. A score of 1 was assigned to each decision the respondent made herself or jointly with her husband/partner. The index was created by summing the scores for the 6 decisions and subdividing them into 3 categories: low (scores 0–1), medium (scores 2–4), and high (scores 5–6). Antepartum and delivery variables related to the last birth included number of ANC visits (0–3 and 4 or more visits), being counseled to develop a birth plan, place of delivery, adequacy of care at the facility where the last birth occurred (defined as having 28 or more minimum facility readiness of care attributes,^34^ as shown in Table 1), mode of delivery (cesarean or vaginal birth), birth companionship during labor and/or delivery in a health facility, satisfaction with delivery care, and frequency of radio listening. The birth-related variables included birth outcome (live or stillbirth), pregnancy intendedness, and experience of postpartum complications.

Because we wanted to examine the adequacy of place of delivery and its effect on PNC, we created a combined 4-level variable (hospital/health center with adequate readiness, hospital/health center with less than adequate readiness, dispensary, and home/on the way) that captured readiness of various facility types where women reported having received delivery care for their last birth. Adequate readiness was defined as having 28 or more attributes present. Because less than 1% of women delivered in dispensaries with adequate readiness, we did not differentiate dispensary delivery by facility readiness.

#### Community-Level Characteristics

We included the following community-level characteristics associated with each PSU: area of residence (rural or urban),^36^ district (Kigoma urban, Kigoma rural, Kibondo, and Kasulu), community-level socioeconomic status (SES), distance to nearest facility providing maternity care, presence of a facility with adequate readiness (i.e., facility with at least 28 readiness attributes) within 5 km and within 5–10 km, community perceptions about quality at the nearest facility, and community perceptions toward delivering in a health facility (used as a proxy for community attitudes toward health care utilization).

Distance to the nearest facility providing maternity care and proximity of a health facility with adequate readiness were calculated by estimating travel distances using roads accessible to walking from the center of each community to each health facility in the region.^32^ We obtained the community geocodes from the Tanzania National Bureau of Statistics and verified them during RHS fieldwork; health facilities were geo-located during Kigoma HFA.

Distances from communities to facilities were estimated using a series of cost-friction surface models by various transportation means in AccessMod version 5 (World Health Organization, Geneva, Switzerland), an analytic extension to ArcGIS 10.6.1. Because provision of PNC needs to be highly accessible, we used the travel distance from each community to the nearest facility measured by walking and categorized it as within 5 km, 5.1–10 km, and more than 10 km.

AccessMod and the health facility characteristics were used to estimate if there were any facilities with adequate readiness providing maternity care located within 5 km walking distance from the community center. A similar variable was created to examine presence of facilities with adequate readiness in 5–10 km walking distance.

The community SES and 2 community perceptions variables (perceptions toward delivering in a health facility and perceptions about quality at the nearest facility) were created by aggregating dichotomized individual-level responses to the 2018 RHS in each community and categorizing the resulting aggregate variables as terciles (low [0%–32.9%], middle [33.0%–65.9%], and high [66% or more]). Communities where 66% or more women resided in households with high SES were classified as having high SES, and those with 66% or more respondents who reported that it is very important to deliver in a health facility were classified as communities with favorable perceptions toward facility use. Similarly, communities where 66% of respondents had a poor opinion of quality of care at the nearest health facility were classified as having high poor opinion.

### Analysis

Analyses were conducted by applying SAS procedures for complex survey design. Weights were calculated based on the inverse probability of selection at each level of the data hierarchy and applied in all analyses. Differences in the proportion of individual and community characteristics between PNC users and nonusers were tested using the Rao-Scott chi-square test. Unadjusted odd ratios were calculated using weighted simple logistic regression models to assess the relationship of each individual and community characteristic with the PNC utilization. Variables were included in the logistic regression model if they were conceptually important (age, literacy, parity, pregnancy intendedness and outcome, ANC use, and community SES level) or if the chi-square test met the *P*<.20 level. Because of the hierarchal nature of the RHS and the clustering of responses at different levels, adjusted odds ratios (aOR) and their corresponding 95% confidence intervals (CI) were calculated using a multilevel mixed-effects logistic regression model with random intercepts for each community.

We fitted an empty model (with no predictors) and 3 mixed-effects multilevel logistic regression models to examine the effects of individual (Model 1), community (Model 2), and both individual and community (Model 3) factors on PNC utilization. For each model, we estimated the intraclass correlation coefficient (ICC) that quantifies the proportion of the observed variance in PNC utilization that is attributable to the effect of geographical clustering of households in communities, as compared to the overall variance.

ICC is calculated as:

$$\text{ICC}=\frac{\sigma_{u_{0}}^{2}}{\sigma_{u_{0}}^{2}+\sigma_{e}^{2}}$$

where σ^2^*u*_0_ is the variance of the level-2 residuals and σ^2^*e* is the variance of the level-1 residuals.^40^

We conducted analyses in SAS version 9.4 (SAS Institute, Cary, North Carolina) using procedures for complex survey data and PROC GLIMMIX for multilevel models.^41^^,^^42^ Results were considered significant if *P*<.05.

### Ethical Approval

The study protocol was approved by the Tanzania National Institute for Medical Research, complied with Tanzania procedures for protecting human rights in research, and was deemed non-research by the CDC Human Research Protection Office of the Center for Global Health.

## RESULTS

### Facility Characteristics

In our analysis of facility attributes that are essential for adequate routine obstetric and newborn care, we found that basic infrastructure attributes (electricity, water and sanitation, and private rooms for consultations) and basic equipment for safe delivery and newborn care (gloves, delivery sets, adequate sterilization, blood pressure cuffs, adult stethoscopes, suction equipment, and newborn bag) were present in most delivery facilities (Figure). Almost all facilities had injectable uterotonics (100%), injectable antibiotics (95.9%), magnesium sulfate (99.5%), antihypertensive medication (77.2%), and antibiotic eye ointment for newborns (88.3%). Most facilities had services available 24 hours a day/7 days per week (97.5%). However, few facilities had an oxygen source (23.4%) or a manual vacuum extractor (51.3%), and fewer had an adequate number of beds (19.3%) or access to motorized transport for referrals (13.7%). Most dispensaries and close to half of health centers did not have enough delivery beds as defined by the MOH guidelines^30^ (Table 2). Less common, particularly in dispensaries, were an adequate number of delivery personnel by facility type, per official recommendations,^43^ and training in basic EmONC. Equipment for assisted vaginal delivery was often missing in dispensaries (46.3%) but generally present in health centers (70.4%) and hospitals (100%), while equipment for removal of retained products (manual or electric vacuum aspirator or dilation and curettage kits) was often available (73.8% of dispensaries, 85.2% of health centers, and 100.0% of hospitals had functional equipment). Availability of essential medicines was generally high.

**FIGURE:**
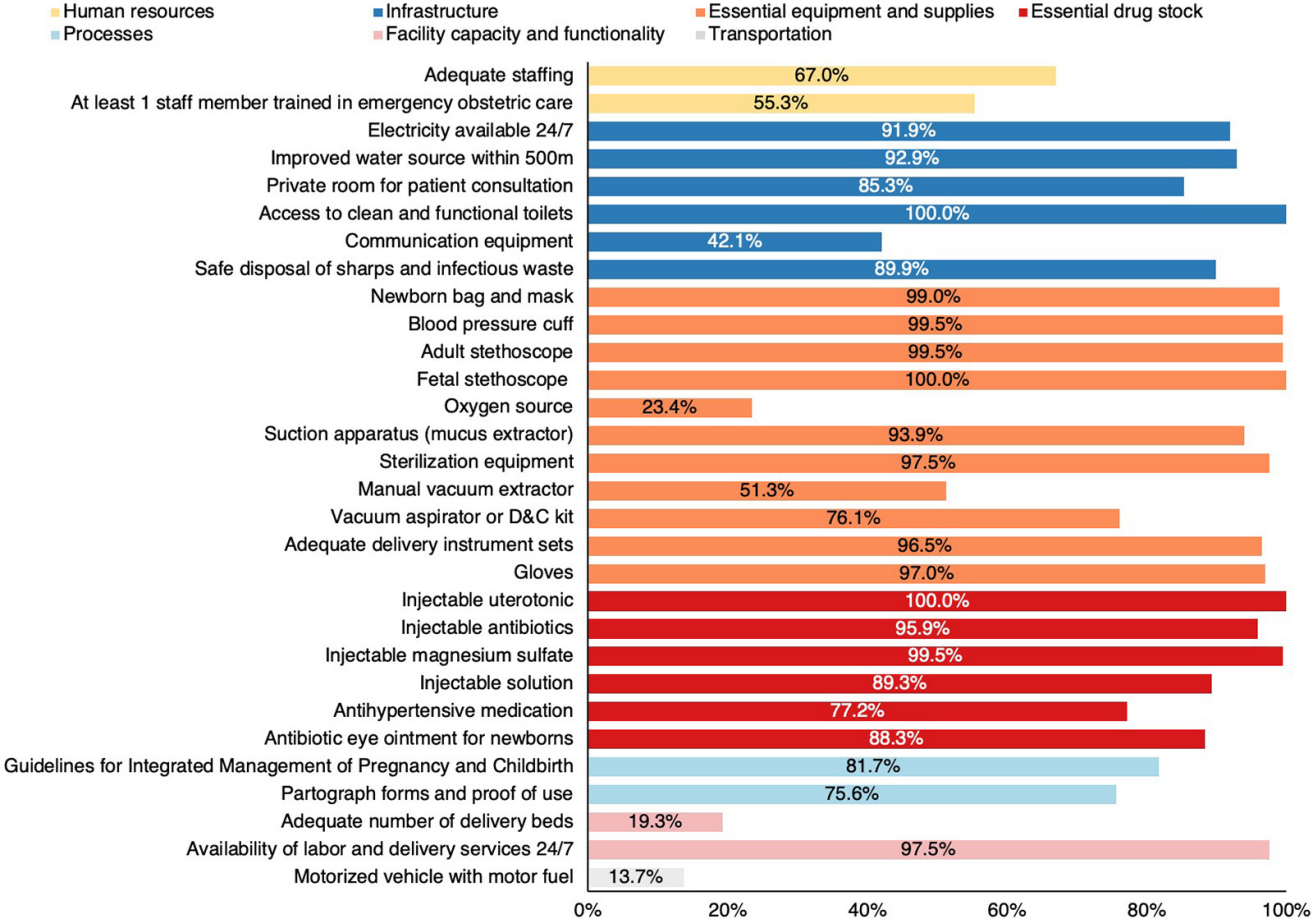
Analysis of Health Facility Attributes That Are Essential for Adequate Routine Obstetric and Newborn Care, Kigoma Region, Tanzania Abbreviation: D&C, dilation and curettage.

**TABLE 2.** Presence of Readiness Attributes in 197 Facilities Providing Maternity Care by Facility Type in Kigoma Region, Tanzania

**Attribute**	**Dispensary, No. (%)**^a^ **(N=164)**	**Health Center, No. (%)**^a^ **(N=27)**	**Hospital, No. (%)**^a^ **(N=6)**
Adequate staffing	112 (68.3)	15 (55.6)	5 (81.3)
At least 1 staff trained in emergency obstetric care	87 (53.1)	18 (66.8)	4 (66.7)
Electricity available 24 hours/day and 7 days/week	153 (93.3)	22 (81.5)	6 (100.0)
Improved water source within 500 m	150 (91.5)	27 (100.0)	6 (100.0)
Private room for patient consultation	137 (83.5)	25 (92.6)	6 (100.0)
Access to clean and functional toilets	164 (100.0)	27 (100.0)	6 (100.0)
Communication equipment	60 (36.6)	18 (66.7)	5 (83.3)
Safe disposal of sharps and infectious waste	144 (87.8)	27 (100.0)	6 (100.0)
Newborn bag and mask	162 (98.8)	27 (100.0)	6 (100.0)
Blood pressure cuff	163 (99.4)	27 (100.0)	6 (100.0)
Adult stethoscope	163 (99.4)	27 (100.0)	6 (100.0)
Fetal stethoscope	164 (100.0)	27 (100.0)	6 (100.0)
Oxygen source	18 (11.0)	22 (81.5)	6 (100.0)
Suction apparatus (mucus extractor)	153 (93.3)	26 (96.3)	6 (100.0)
Sterilization equipment	159 (97.0)	27 (100.0)	6 (100.0)
Manual vacuum extractor for assisted vaginal delivery	76 (46.3)	19 (70.4)	6 (100.0)
Vacuum aspirator or D&C kit	121 (73.8)	23 (85.2)	6 (100.0)
Adequate delivery instrument sets	157 (95.7)	27 (100.0)	6 (100.0)
Gloves	158 (96.3)	27 (100.0)	6 (100.0)
Injectable uterotonic	164 (100.0)	27 (100.0)	6 (100.0)
Injectable antibiotics	157 (95.7)	26 (96.3)	6 (100.0)
Injectable magnesium sulfate	163 (99.4)	27 (100.0)	6 (100.0)
Intravenous solution	145 (88.4)	26 (96.3)	5 (83.3)
Antihypertensive medication	119 (72.6)	27 (100.0)	6 (100.0)
Antibiotic eye ointment for newborns	145 (88.4)	24 (88.9)	5 (88.3)
Guidelines for Integrated Management of Pregnancy and Childbirth	137 (83.5)	19 (70.4)	5 (83.3)
Partograph forms and proof of use	122 (74.4)	21 (77.8)	6 (100.0)
Adequate number of delivery beds	17 (10.4)	15 (55.6)	6 (100.0)
Availability of labor and delivery services 24 hours/day and 7 days/week	159 (97.0)	27 (100.0)	6 (100.0)
Motorized vehicle with motor fuel	6 (3.7)	18 (66.7)	3 (50.0)
Quality index of 28 attributes or more (highest tercile)	1 (0.61)	11 (40.7)	5 (83.3)
Mean number of attributes (SD)	23.4 (23.0–23.7)	26.5 (25.5–27.4)	28.3 (26.9–29.8)

Abbreviations: D&C, dilation and curettage; SD, standard deviation.

^a^ Counts and percentages represent unweighted frequencies of a specified element within a facility type.

Access to motorized vehicles with fuel for referrals was moderate in hospitals and health centers (50.5% and 66.7%) and very low in dispensaries (3.7%). Hospitals had the highest readiness, with a mean of 28.3 facility attributes, followed by health centers (25.5 mean number of attributes) and dispensaries (23.4 mean number of attributes).

### Individual and Community Characteristics and PNC Use

The PNC utilization rate among women in Kigoma Region was 15.9% (683 women; 95% CI=13.5, 18.5). Almost 40% were younger than age 25 years (Table 3). Almost two-thirds (63.8%) of women were literate. Most women were married (86.3%), reported not working outside of the home (68.5%), and resided in households with middle (34.5%) or high (29.8%) wealth. Only one-third (34.3%) of women reported high decision-making autonomy. Women with high parity (5 or more previous live births) represented 38.2% of the sample. Over one-half (58.2%) of women had at least 4 ANC visits for the most recent birth. Almost all women were counseled by a health provider during pregnancy to have a birth plan (91.0%).

**TABLE 3.** Selected Individual and Community Characteristics and Use of PNC After Last Birth Among Women Aged 15–49 Years in Kigoma Region, Tanzania

**Individual Characteristic**	**All Women, No. (%)**^a^ (N=4,353)	**Used PNC, No. (%)**^a^ (N=683)	**Did Not Use PNC, No. (%)**^a^ (N=3,670)	***P* Value** ^b^
Maternal age at birth, years				.46
Under 25	1719 (39.8)	286 (41.1)	1,433 (39.6)	
25–34	1,712 (39.4)	252 (37.1)	1,460 (39.8)	
35–49	922 (20.8)	145 (21.8)	777 (20.6)	
Literacy				.60
No	1,589 (36.2)	242 (35.3)	1,347 (36.4)	
Yes	2,764 (63.8)	441 (64.7)	2,323 (63.6)	
Work status				.17
Does not work outside home	2,982 (68.5)	450 (64.9)	2,532 (69.3)	
Works outside home	1,371 (31.5)	233 (35.1)	1,138 (30.7)	
Wealth tercile				.06
Low	1,601 (35.7)	224 (30.2)	1,377 (36.8)	
Middle	1,575 (34.5)	258 (35.9)	1,317 (34.2)	
High	1,177 (29.8)	201 (33.9)	976 (29.0)	
Current marital status				.05
Married	3,752 (86.3)	600 (88.9)	3,152 (85.8)	
Not married	601 (13.7)	83 (11.1)	518 (14.2)	
Decision-making index				<.0001
Low/medium	2,840 (65.7)	384 (55.9)	2,456 (67.7)	
High	1,513 (34.3)	299 (44.1)	1,214 (32.3)	
Parity				.49
1 child	847 (19.7)	148 (20.6)	699 (19.5)	
2–4 children	1,810 (42.1)	282 (44.3)	1,528 (41.8)	
5 or more children	1,696 (38.2)	253 (35.1)	1,443 (38.7)	
ANC utilization				.33
0–3 visits	1,842 (41.8)	302 (43.8)	1,540 (41.3)	
4 or more visits	2,511 (58.2)	381 (56.2)	2,130 (58.7)	
Counseled for birth plan during pregnancy				.0144
No	382 (9.0)	46 (6.7)	336 (8.8)	
Yes	3,971 (91.0)	637 (93.3)	3,334 (91.2)	
Place of delivery and its adequacy^c^				<.0001
Home/on the way	1,261 (22.4)	98 (13.2)	916 (24.2)	
Dispensary any readiness	1,515 (34.5)	241 (36.1)	1,274 (34.2)	
Hospital/HC with less than adequate readiness	563 (11.7)	83 (10.5)	480 (11.9)	
Hospital/HC with adequate readiness	1,515 (31.4)	261 (40.2)	1,000 (29.7)	
Mode of delivery				<.0001
Normal vaginal	4,150 (95.0)	619 (89.4)	3,531 (96.1)	
Cesarean	203 (5.0)	64 (10.6)	139 (3.9)	
Companionship at birth				.0104
No companion during labor or delivery	3,021 (68.9)	430 (61.8)	2,591 (70.3)	
Companion during labor or delivery	1,332 (31.1)	253 (38.2)	1,079 (29.7)	
Pregnancy intendedness				.2463
Intended	3,174 (73.2)	518 (75.5)	2,656 (72.8)	
Unintended	1,179 (26.8)	165 (24.5)	1,014 (27.2)	
Birth outcome				.6074
Live birth	4,302 (98.9)	675 (98.7)	3,627 (98.9)	
Stillbirth	51 (1.1)	8 (1.3)	43 (1.1)	
Postpartum complications				.1342
Absent	2,651 (60.9)	444 (64.7)	2,207 (60.1)	
Present	1,702 (39.1)	239 (35.3)	1,463 (39.9)	
Satisfaction with delivery services				.0002
Not satisfied/did not deliver in facility	1,099 (24.5)	111 (15.2)	988 (26.4)	
Satisfied/somewhat satisfied	3,254 (75.5)	572 (84.8)	2,682 (73.6)	
Frequency of radio listening				.1128
Never	1,975 (45.3)	276 (41.6)	1,699 (46.1)	
Sometimes	2,378 (54.7)	407 (58.4)	1,971 (53.9)	
**Community Characteristic**				
Area of residence				.12
Rural	3,752 (84.5)	565 (80.9)	3,187 (85.2)	
Urban	601 (15.5)	118 (19.1)	483 (14.8)	
Community socioeconomic status				
Low	1,683 (39.8)	265 (35.5)	1,418 (40.6)	.50
Middle	1,453 (31.2)	218 (33.2)	1,235 (30.8)	
High	1,217 (29.0)	200 (31.3)	1,017 (31.3)	
District				<.0001
Kigoma Rural	1,315 (27.4)	92 (11.2)	1,223 (30.5)	
Kigoma Urban	388 (11.2)	77 (13.8)	311 (10.6)	
Kibondo	1,031 (23.3)	198 (26.3)	833 (22.7)	
Kasulu	1,619 (38.2)	316 (48.7)	1,303 (36.2)	
Distance to nearest facility providing maternity care^d^				.10
0–5 km	3,421 (76.7)	575 (82.7)	2,846 (75.6)	
5.1–10 km	490 (12.0)	83 (12.4)	407 (11.9)	
More than 10 km	442 (11.3)	25 (4.9)	417 (12.5)	
Adequate facility^c^^,d^ within 5 km				.0031
No	3,632 (81.9)	516 (74.5)	3,116 (83.3)	
Yes	721 (18.1)	167 (25.5)	554 (16.7)	
Adequate facility^c^^,d^ within 5–10 km				.4237
No	3,670 (84.1)	489 (72.2)	2,818 (75.5)	
Yes	683 (15.9)	194 (27.8)	852 (24.5)	
Community perception that quality of care at nearest health facility is poor^e^				.0844
Low	1,399 (32.1)	245 (34.6)	1,154 (31.7)	
Middle	1,571 (38.9)	287 (44.3)	1,284 (37.8)	
High	1,383 (29.0)	151 (21.1)	1,232 (30.5)	
Community perception that it is very important to deliver in a health facility^e^				.0305
Low	1,655 (41.4)	196 (31.8)	1,459 (43.2)	
Middle	1,422 (33.3)	259 (38.4)	1,163 (32.4)	
High	1,276 (25.2)	228 (29.8)	1,048 (24.4)	

Abbreviations: ANC, antenatal care; HC, health center; PNC, postnatal care.

a Frequencies in the study population are presented as unweighted counts and weighted percentages. Percentages may not sum up to 100 due to rounding.

b *P* values were derived from Rao-Scott chi-square test.

c Facility adequacy was estimated using the World Health Organization guidelines of assessing facility readiness for routine maternity care.^34^ Each facility was assessed based on presence or absence of 30 attributes necessary for performing routine maternity services. We created an index score ranging from 0 to 30 based on the presence of each attribute. Facilities that had a score in the highest decile (i.e., having 28 or more attributes) were classified as having adequate readiness for routine maternal care services. The other facilities were classified as having “less than adequate readiness.” There were 68 women who delivered in the region in small-volume dispensaries that were not included in the health facility assessment. Their delivery facilities were classified as having less than adequate readiness based on inputs from health managers in the region.

d Distance estimated using ArcGIS models for traveling by roads or paths accessible to walking.

e Created by aggregating dichotomized individual level responses in each community and categorizing the resulting aggregate variables as terciles (low [0–32.9%], middle [33.0–65.9%], and high [66% or more]).

Most women (77.6%) delivered in a health facility (hospital, health center, or dispensary), including 5% who had a cesarean delivery. Less than one-third (31.1%) of women had a companion during labor or delivery. Almost three-quarters (73.2%) of women reported that the last birth was intended. Three-quarters (75.5%) of women were satisfied or somewhat satisfied with their delivery care.

Most women (84.5%) in the sample resided in rural communities, and most women had low (39.8%) or middle (31.2%) socioeconomic status. Three-quarters (76.7%) resided in communities with a health facility nearby (within 0–5 km), with an additional 12% residing in communities with the nearest facility within 5.1–10 km. However, only 18.1% of women resided in communities that were within 0–5 km of at least 1 health facility with adequate readiness, and 15.9% resided within 5.1–10 km of a facility with adequate readiness.

Bivariate analyses (Table 4, first panel) showed greater odds of PNC utilization among women residing in households with middle or high wealth, married women, those with high decision-making autonomy, and those who received counseling about having a birth plan, delivered in an adequate hospital or health center as opposed to delivering at home, had a cesarean delivery, had a companion at birth, or were satisfied with the facility care at birth. Greater odds of PNC were also found among those with urban residence, residence in Kigoma urban, Kasulu, or Kibondo district as opposed to Kigoma rural district, residence in a community where the nearest health facility was within 5 km or where at least 1 adequate facility was within 0–5 km, and residence in a community where a higher proportion of women stated that it is very important to deliver in a health facility. Residing in a community with higher perception that quality of care at the nearest facility was poor was associated with lower PNC utilization.

**TABLE 4.** Unadjusted and Adjusted Statistical Associations of Individual and Community Characteristics and PNC Use in Kigoma Region Tanzania Based on 3 Random Intercept Models

	Unadjusted			Model 1 Individual Characteristics			Model 2 Community Characteristics			Model 3 Individual and Community		
Individual Characteristic	OR	95% CI	*P* Value	aOR	95% CI	*P* Value	aOR	95% CI	*P* Value	aOR	95% CI	*P* Value
Maternal age at birth, years												
Under 25	1.00 (ref)			1.00 (ref)						1.00 (ref)		
25–34	0.90	0.74–1.09	.2074	0.92	0.72–1.18	.4896				0.91	0.71–1.17	.4727
35–49	1.02	0.88–1.30	.5028	1.02	0.67–1.57	.9234				1.01	0.65–1.56	.981
Literacy												
No	1.00 (ref)			1.00 (ref)						1.00 (ref)		
Yes	1.05	0.85–1.25	.613	0.84	0.68–1.04	.1027				0.83	0.67–1.02	.0767
Work status												
Does not work outside home	1.00 (ref)			1.00 (ref)						1.00 (ref)		
Works outside home	1.22	0.91–1.63	.1760	1.02	0.75–1.40	.8992				0.99	0.73–1.35	.9456
Wealth tercile												
Low	1.00 (ref)			1.00 (ref)						1.00 (ref)		
Middle/high	1.34	1.05–1.72	.0207^a^	1.25	1.00–1.59	.0628				1.25	0.99–1.59	.0705
Current marital status												
Married	1.33	1.03–1.76	.0472^a^	1.05	0.78–1.41	.7436				1.07	0.80–1.45	.6709
Not married	1.00 (ref)			1.00 (ref)						1.00 (ref)		
Decision-making index												
Low/medium	1.00 (ref)			1.00 (ref)						1.00 (ref)		
High	1.65	1.29–2.12	.0001^a^	1.57	1.12–2.19	.0093^a^				1.56	1.11–2.17	.011^a^
Parity												
1 child	1.00 (ref)			1.00 (ref)						1.00 (ref)		
2–4 children	1.01	0.69–1.47	.59	1.00	0.72–1.40	.9865				1.01	0.72–1.42	.9297
5 or more children	0.86	0.66–1.13	.1473	0.92	0.60–1.43	.725				0.95	0.61–1.48	.8302
ANC utilization												
0–3 visits	1.00 (ref)			1.00 (ref)						1.00 (ref)		
4 or more visits	0.90	0.73–1.11	.328	0.84	0.68–1.04	.124				0.85	0.68–1.05	.1364
Counseled for birth plan												
No	1.00 (ref)			1.00 (ref)						1.00 (ref)		
Yes	1.46	1.09–2.00	.0114^a^	1.47	1.00–2.19	.0629				1.40	0.93–2.12	.1089
Place of delivery and its adequacy^b^												
Home/on the way	1.00 (ref)			1.00 (ref)						1.00 (ref)		
Dispensary, any readiness	1.94	1.24–3.01	.169	0.86	0.37–2.00	.7315				0.79	0.34–1.86	.5969
Hospital/HC with less than adequate readiness	1.61	1.02–2.55	.7793	0.98	0.41–2.36	.9761				0.87	0.36–2.09	.8869
Hospital/HC with adequate readiness	2.48	1.62–3.81	.0003^a^	1.17	0.51–2.66	.7161				1.06	0.46–2.48	.7509
Mode of delivery												
Normal vaginal	1.00 (ref)			1.00 (ref)						1.00 (ref)		
Cesarean	2.88	1.95–4.27	<.0001^a^	2.29	1.50–3.49	.0002^a^				2.27	1.47–3.48	.0002^a^
Companionship at birth												
No companion during labor or delivery	1.00 (ref)			1.00 (ref)						1.00 (ref)		
Companion during labor or delivery	1.46	1.12–1.91	.0059^a^	1.54	1.16–2.04	.0024^a^				1.57	1.19–2.07	.0013^a^
Pregnancy intendedness												
Intended	1.16	0.91–1.47	.2463	1.11	0.87–1.42	.4081				1.11	0.87–1.42	.4086
Unintended	1.00 (ref)			1.00 (ref)						1.00 (ref)		
Birth outcome												
Live birth	0.82	0.38–1.78	.6074	1.13	0.57–2.24	.7199				1.04	0.53–2.06	.9057
Stillbirth	1.00 (ref)			1.00 (ref)						1.00 (ref)		
Postpartum complications												
Absent	1.00 (ref)			1.00 (ref)						1.00 (ref)		
Present	0.82	0.64–1.06	.1342	0.85	0.64–1.14	.2755				0.89	0.66–1.19	.4184
Satisfaction with delivery services												
Not satisfied/did not deliver in facility	1.00 (ref)			1.00 (ref)						1.00 (ref)		
Satisfied/somewhat satisfied	2.00	1.39–2.87	.0002^a^	1.32	0.64–2.71	.4519				1.33	0.64–2.71	.4493
Frequency of radio listening												
Never	1.00 (ref)			1.00 (ref)						1.00 (ref)		
Sometimes	1.21	0.96–1.54	.1128	1.09	0.87–1.37	.4457				1.06	0.84–1.32	.6263
**Community Characteristic**												
Area of residence												
Rural	1.00 (ref)						1.00 (ref)			1.00 (ref)		
Urban	1.79	1.15–2.79	.0099^a^				1.51	0.78–2.96	.2233	1.34	0.67–2.76	.4156
Community socioeconomic status												
Low	1.00 (ref)						1.00 (ref)			1.00 (ref)		
Middle	0.92	0.54–1.58	.769				1.03	0.60–1.77	.90	0.87	0.49–1.53	.6294
High	1.31	0.81–2.12	0.276				0.79	0.47–1.36	.4102	0.71	0.41–1.23	.2163
District												
Kigoma Rural	1.00 (ref)						1.00 (ref)			1.00 (ref)		
Kigoma Urban	3.61	2.00–6.51	<.0001^a^				0.80	0.30–2.12	.657	0.88	0.32–2.44	.8148
Kibondo	2.94	1.66–5.24	.0002^a^				2.03	1.03–4.01	.0407^b^	1.80	0.88–3.67	.1096
Kasulu	3.68	2.38–5.70	<.0001^a^				3.15	1.92–5.17	<.0001^c^	3.28	1.94–5.52	<.0001^a^
Distance to nearest facility with maternity care^c^												
0–5 km	3.48	1.38–8.69	.0080^a^				2.01	0.97–4.17	.0598^a^	1.95	0.92–4.16	.0868
5.1–10 km	2.72	0.83–8.94	.0988				2.06	0.89–4.80	.0936^a^	2.13	0.90–5.05	.0876
More than 10 km	1.00 (ref)						1.00 (ref)			1.00 (ref)		
Adequate facility ^b^^,c^ within 5 km												
Absent	1.00 (ref)						1.00 (ref)			1.00 (ref)		
Present	2.31	1.53–3.49	<.0001^a^				2.44	1.37–4.37	.0026^c^	2.15	1.06–3.88	.032^a^
Adequate facility ^b^^,c^ within 5–10 km												
Absent	1.00 (ref)						1.00 (ref)			1.00 (ref)		
Present	1.30	0.82–2.06	.2698				0.98	0.57–1.69	.9496	1.04	0.59–1.81	.8878
Community perception that quality of care at nearest health facility is poor^d^												
Low	1.00 (ref)						1.00 (ref)			1.00 (ref)		
Middle	1.05	0.67–1.65	.8365				1.16	0.76–1.78	.4915	0.94	0.57–1.56	.3356
High	0.52	0.31–0.87	.0128^a^				0.88	0.53–1.45	.604	1.24	0.80–1.91	.818
Community perception that it is very important to deliver in a health facility^d^												
Low	1.00 (ref)						1.00 (ref)			1.00 (ref)		
Middle	1.82	1.08–3.08	.0252^a^				1.26	0.77–2.06	.3528	1.26	0.74–2.16	.3935
High	1.93	1.13–3.31	.0169^a^				1.24	0.74–2.08	.4058	1.25	0.75–2.07	.3919
Variance (SE)				1.24 (0.21)			0.85 (0.16)			0.91 (0.17)		
ICC				27%			21%			22%		

Abbreviations: aOR, adjusted odds ratio; ANC, antenatal care; AVD, assisted vaginal delivery; CI, confidence interval; ICC, intraclass correlation coefficient; OR, odds ratio; PNC, postnatal care; ref., reference group; SE, standard error.

a Significant at *P* value <.05.

b Facility adequacy was estimated using the World Health Organization guidelines of assessing facility readiness for routine maternity care.^34^ Each facility was assessed based on presence or absence of 30 attributes necessary for performing routine maternity services. We created an index score ranging from 0 to 30 based on the presence of each attribute. Facilities that had a score in the highest decile (i.e., having 28 or more attributes) were classified as having adequate readiness for routine maternal care services. The other facilities were classified as having less than adequate readiness. There were 68 women who delivered in the region in small-volume dispensaries that were not included in the health facility assessment. Their delivery facilities were classified as having less than adequate readiness based on inputs from health managers in the region.

c Distance estimated using ArcGIS models for travelling by roads or paths accessible to walking.

d Created by aggregating dichotomized individual level responses in each community and categorizing the resulting aggregate variables as terciles (low [0%–32.9%], middle [33.0%–65.9%], and high [≥66%]).

### Multilevel Analyses

When we examined the association between individual characteristics and PNC in health facilities (Model 1), we found that having high decision-making autonomy, having companionship at birth, and having a cesarean delivery were significantly associated with PNC at *P*<.05. The ICC in Model 1 indicated that 27% of the variation in women’s PNC use was attributable to differences across communities. The examination of community variables (Model 2) indicated that women from Kibondo and Kasulu districts and those residing in communities with a facility with high readiness were significantly associated with PNC use. Residing in communities with the nearest facility within 0–10 km distance was not significant in PNC use. The ICC in Model 2 implied that differences between communities account for 21% of the variation in women’s PNC use.

The multilevel random intercept logistic regression model fitted with individual and community predictors of PNC use (Model 3) showed greater odds of PNC utilization among women with high decision-making autonomy (aOR: 1.56; 95% CI=1.11, 2.17). Women who had a birth companion during labor or delivery were 1.6 times (aOR 1.57; 95% CI=1.19, 2.07) more likely to seek facility PNC than women who had no birth companion during labor or delivery. Those who had a cesarean delivery were 2.3 times (aOR 2.27; 95% CI=1.47, 3.48) more likely to seek facility PNC than women who delivered vaginally. Being literate, living in a household with middle or high wealth, and being counseled during pregnancy to develop a birth plan were not associated with facility PNC use, nor were other demographic and pregnancy-related variables (age, marital status, parity, pregnancy intendedness, ANC use, place of delivery, pregnancy outcome, experience of postpartum complications, satisfaction with delivery services, and media exposure).

Women residing in Kasulu district had the highest odds of utilizing PNC (aOR: 3.28; 95% CI=1.94, 5.53) in Kigoma Region. Proximity of the nearest facility alone (travel distance by walking of less than 5 km or 5.1–10 km) was not associated with PNC use. However, women who lived within a 5 km travel distance to a facility with adequate readiness were 2 times more likely to use facility PNC (aOR: 2.15; 95% CI=1.05, 3.85) than women residing in communities without a nearby adequate facility. Those residing in communities within 5.1–10 km travel distance to a facility with adequate readiness did not have higher odds of facility PNC (aOR: 1.04; 95% CI=0.59, 1.81) compared to women residing in communities with more distant adequate facilities. Community socioeconomic status, high perception that quality of care at the nearby facility was poor, and high perceptions toward facility use were not associated with increased PNC use.

The ICC estimated from the empty model was 28% (data not shown), indicating that there was substantial variability in PNC use among communities. The inclusion of the community covariates reduced the ICC to 21%, suggesting that these variables explain a quarter of the between-communities variability in the odds of facility PNC use.

## DISCUSSION

PNC provides an opportunity to prevent, detect, and address postpartum maternal complications and is critical in sub-Saharan Africa, where most maternal deaths occur after birth.^7^ Access and use of PNC services is low in Kigoma Region across all women, including those who experienced postpartum complications. Our study found lower utilization of PNC in Kigoma Region (16%) than reported nationally (36%).^3^ The lower estimate in our study may be explained by the exclusion of PNC experienced by women before their discharge from their delivery facilities. Low rates of PNC use in Tanzania are in sharp contrast with high ANC and delivery care in health facilities^3^ and with national^6^ and global recommendations.^44^

Our study found lower utilization of PNC in Kigoma Region (16%) than reported nationally (36%).

Improving the pregnancy continuum of care requires having a critical understanding of facilitators and barriers to PNC. Controlling for all other factors, we found that several individual and community factors were associated with PNC, including decision-making autonomy, having a birth companion for support during labor and delivery, experiencing a cesarean delivery, district of residence, and presence of an adequate health facility within 5 km travel distance.

Increased PNC among women with high decision-making autonomy and companionship at delivery found in our study is consistent with previous research indicating that social support from family and friends, including spouses, is associated with maternal health care-seeking behaviors.^45^^,^^46^ Giving birth in a health facility in the presence of a birth companion was associated with higher facility PNC use in our study. Facility policies that promote and facilitate birth companionship are beneficial for emotional support at birth^47^^,^^48^ and early PNC initiation.^14^ Unlike other studies suggesting that a greater level of attendance at ANC (4 or more visits) and satisfaction with facility delivery care may improve the likelihood of seeking future care in health facilities,^14^^,^^49^^–^^51^ we did not find an association of such experiences with PNC. Similarly, we did not find that literacy, household wealth, and parity were significant predictors of PNC use. Consistent with findings from other studies,^17^^,^^18^ women with cesarean deliveries (necessary to address maternal or fetal complications) were more likely to seek PNC than women with vaginal deliveries. However, experience of postpartum complications was not a predictor of PNC use, suggesting that women with cesarean deliveries may have sought PNC either for follow-up care of their ante- or intrapartum complications or for care of the surgical incision.

Women residing in proximity (within 5 km) to a health facility with adequate readiness were twice as likely to use facility PNC as women residing near a facility with less adequate services, suggesting that the geographic accessibility to an adequate facility may affect a woman’s decision to use PNC services, independent of other factors. Proximity of a facility with adequate readiness was 1 of the strongest community factors associated with PNC use (*P*<.01 in the community model and <.05 in the full model). Similar to our findings, while not specific to PNC, a systematic review of childbirth attendance strategies identified that proximity to adequate services was associated with lower maternal mortality in low-income settings.^52^

Women may choose to receive PNC at the facility closest to them regardless of the facility’s adequacy/readiness, especially following normal vaginal deliveries. Proximity (within 5 km) to a health facility alone increased the unadjusted (*P*=.0080) and adjusted odds of PNC (*P*=.0868). The odds of seeking PNC decreased when travel distances to an adequate facility exceeded 5 km. While the strength of association was lower in our study than that shown in several studies examining determinants of facility delivery,^22^^,^^53^^,^^54^ it reaffirms that reducing distance to care may promote PNC attendance at a facility, regardless of the facility’s attributes.

Our study is unique in measuring the travel distance from community to an adequate facility because we examined the influence on PNC uptake of both distance and availability of essential attributes of care. Previous studies examining the relationship between health service utilization and facility adequacy and distance have examined these covariates separately and produced varied results. Some researchers found that obstetric functionality at the nearest facility was not associated with use, except when the closest facility offered suboptimal EmONC,^22^ while others showed that facility characteristics and distance are important drivers of using health care services.^26^^,^^27^^,^^55^ However, all but 1^27^ of these studies used ecological linkages to investigate whether distance and quality of care influenced the place women sought maternity services; the majority used Euclidean, straight-line distances between communities and health facilities that do not account for the difficulties of the terrain; and all relied on just a handful of attributes to assess the adequacy of services. Moreover, these studies focused on the role of distance and quality in accessing delivery, family planning, or child health care services not PNC services.

Our study is unique in measuring the travel distance from community to an adequate facility because we examined the influence on PNC uptake of both distance and of availability of essential attributes of care.

Unlike other studies that examined perceived quality of care and found that it may guide a woman’s decision to seek facility delivery care,^23^^,^^53^^,^^54^ we did not find an association between perceived poor quality received at delivery and PNC utilization in the full model. Although quality of care is complex and difficult to measure,^56^ evidence shows that poor quality of care at health facilities is a leading factor in high rates of morbidity and mortality in women and infants.^57^

Donabedian’s model for health care quality cites structure, process, and outcome as quality domains.^58^ Facility adequacy measured in our study captures many elements of structure and process but does not capture health providers’ behaviors, their training and knowledge, the care they administer, or adequacy of patient-provider interactions. It is possible that the observed lack of an association between reported/perceived quality of care at birth or perceived quality of the nearby facility and PNC utilization may be due to using a readiness index that does not capture all quality dimensions. Future studies may consider using readiness in conjunction with additional measures of quality to provide a better understanding of how a facility’s quality affects its use.

### Strengths and Limitations

Our study is among the few to directly link individual women with the facilities they have been using and with the facilities proximal to their communities. Many studies have largely used surveys such as the Multiple Indicator Cluster Surveys or Demographic Health Surveys to ecologically link household and facility data; however, these studies introduce limitations such as linking individual women to facilities that they did not use.^28^ Our methodological approach allowed us to directly examine the association between quality of care at birth and PNC uptake, even when women bypassed their closest facility. It also allowed for the examination of proximity to adequate facilities and PNC use after considering individual, community, and facility characteristics. Creating a community-level variable to capture the readiness of the proximal health facilities allowed us to explore the potential effect of facility characteristics on PNC uptake for all women, not just on those who delivered in a health facility. Lastly, due to the HFA data collection being performed by an outside organization instead of by health facility staff, the potential for social desirability and response bias in our study was reduced, though it may still affect responses.

Despite its strengths, this study came with limitations. The cross-sectional design of our study does not allow us to draw cause-effect conclusions. Survey data were self-reports from mothers within 2–3 years preceding the survey, and this could be a potential source of recall and misclassification bias. Limiting the analysis to the last birth in the interval may have reduced this bias. Investigation of the quality of the PNC services that women received was outside the scope of our study. However, given that a wide range of services for both mothers and newborns are offered during PNC visits, further research would be needed to better understand PNC use in Kigoma Region. This analysis is representative of Kigoma Region and does not allow for generalizing outside of our population. Facility readiness mostly reflects the situation in the last 12 months, and it may have been lower or higher at the time the women in our sample used delivery and PNC services. However, the community variable was created independent of individual experiences. Having the most recent characterization of the proximal facility may be beneficial, as interventions and women receiving them may be influenced by the “current” facility readiness. Finally, this study only included women who received PNC at a health facility, excluding a subset of women who chose to receive PNC at home.

## CONCLUSION

Although PNC is considered an essential component in the continuum of care, utilization rates remain low in Tanzania. This study emphasizes the need to examine health care-seeking behavior through the lens of individual, community, and health facility characteristics. Physical proximity to a health facility with adequate infrastructure, equipment, and workforce increased the odds of PNC use. Improving access to adequate health facilities may provide an essential underpinning for increasing health care utilization. Future research is needed to better understand the role of other quality of care aspects, such as management practices, social support, and providers’ knowledge and interpersonal skills, in encouraging women to seek facility-based health care.

## Supplementary Material

GHSP-D-22-00502-supplement.pdf

## Notes

**Peer Reviewed**

**First published online:** August 11, 2023.
