# Supplementary material for: Individual, Community, and Health Facility Predictors of Postnatal Care Utilization in Rural Tanzania: A Multilevel Analysis
Source: Glob Health Sci Pract. 2023 Aug 28;11(4):e2200502. doi: 10.9745/GHSP-D-22-00502 (PMC10461704; doi:10.9745/GHSP-D-22-00502)
Supplement: GHSP-D-22-00502-supplement.pdf [file GHSP-D-22-00502-supplement.pdf]

Supplement to: Serbanescu F, Abeysekara P, Ruiz A, et al. Individual, community, and health facility predictors of postnatal care utilization in rural Tanzania: a multilevel analysis. *Glob Health Sci Pract*. 2023;11(4):e2200502.
https://doi.org/10.9745/GHSP-D-22-00502

Supplement. Operational definitions of facility readiness attributes in the 2018 Health Facility Assessment (HFA) in Kigoma, Tanzania

Facility Readiness Attribute	Definition
Human Resources	
**Adequate number of delivering personnel, per Ministry of Health staffing guidelines**	Reported number of delivery personnel is in accordance with the national staffing guidelines for hospitals, health centers and dispensaries[26]
**At least one staff member trained in basic emergency obstetric care (EmONC)**	At least one staff member providing maternity services was trained in basic EmONC in the last 2–3 years (training was introduced in 2012 and is required by the national guidelines for all delivering personnel)
Infrastructure	
**Electricity available 24/7**	Reported routine availability of electricity for lights and communication (at a minimum) from any power source during normal working hours and at night
**Improved water source within 500m of facility**	Observed availability and reported functionality of improved water source (piped, public tap, standpipe, tube well/borehole/protected dug well, protected spring or rainwater collection) on the premises or within 500m of facility
**Private room for patient consultation**	Observed availability of private room or screened off area in the main service area
**Access to clean and functioning toilets**	Observed availability of improved sanitation facilities for clients (flush/pour flush to sewer system or to septic tank or to pit latrine, pit latrine with slab, covered)
**Communication equipment**	Observed availability of functioning short-wave radio equipment or facility phone (landline or cellular); private phones used by staff to communicate emergencies and referrals were not included, as no facility reimburses personnel for cost of phone calls using private phones
**Safe disposal of sharps and infectious waste**	Observed availability of safe disposal of sharps and of infectious and biological waste and an environment free of waste
Essential Equipment and Supplies	
**Newborn bag and mask**	Observed availability and reported functionality of newborn bag and mask in the delivery area
**Blood pressure cuff**	Observed availability and reported functionality of digital blood pressure machine or manual sphygmomanometer
**Adult stethoscope**	Observed availability and reported functionality of adult stethoscope in the labor and delivery area
**Fetal stethoscope**	Observed availability and reported functionality of fetal stethoscope in the labor and delivery area
**Oxygen source**	Oxygen source present in the delivery area
**Suction apparatus (mucus extractor)**	Observed availability and reported functionality of suction bulb (single use or re–sterilizable) or electric suction apparatus with catheter in the delivery area
**Sterilization equipment**	Observed availability and reported functionality of sterilization of equipment: dry heat, steaming or boiling

Supplement to: Serbanescu F, Abeysekara P, Ruiz A, et al. Individual, community, and health facility predictors of postnatal care utilization in rural Tanzania: a multilevel analysis. *Glob Health Sci Pract*. 2023;11(4):e2200502. https://doi.org/10.9745/GHSP-D-22-00502

Facility Readiness Attribute	Definition
**Vacuum aspirator or D&C kit**	Observed availability and reported functionality of vacuum aspirator or D&C kit reported functioning in service area
**Manual vacuum extractor**	Observed availability and reported functionality of manual vacuum extractor in the delivery area
**Adequate delivery instrument set**	Observed availability of at least one complete delivery set (including clamp, scissor/blade, suture material, needle and needle holder) present in the delivery area
**Gloves**	Observed availability of disposable latex gloves present in the delivery area (from box, or delivery kit or delivery set)
Essential Drug Stock	
**Injectable uterotonic**	Oxytocin or other injectable uterotonic present in the labor and delivery area or in the main location in the facility where medicines are routinely stored
**Injectable antibiotics**	Injectable antibiotic (ampicillin/gentamicin OR ceftriaxone) present in the labor and delivery area or in the main location of the facility where medicines are routinely stored
**Injectable magnesium sulphate**	Injectable magnesium sulphate present in the labor and delivery area or in the main location of the facility where medicines are routinely stored
**Intravenous solution**	Intravenous solution (saline or Ringer's lactate and Dextrose 5%) with infusion set present in the labor and delivery area or in the main location of the facility where medicines are routinely stored
**Antihypertensive medication**	Antihypertensive drugs (hydralazine, labetalol, methyldopa or nifedipine) present in the labor and delivery area or in the main location of the facility where medicines are routinely stored
**Antibiotic eye ointment for newborns**	Tetracycline or other antibiotic eye ointment for newborn present in the delivery area or in the main location of the facility where medicines are routinely stored
Processes	
**Guidelines for integrated management of pregnancy and childbirth (IMPAC)**	IMPAC guidelines observed in the labor and delivery area
**Partograph forms and proof of use**	Blank partographs present in the labor and delivery area AND current/recent delivering women had partograph completed
Facility capacity and functionality	
**Adequate number of delivery beds**	Delivery beds present in delivery area; facility has the adequate number of delivery beds in accordance with the Tanzania guidelines
**Availability of labor & delivery services 24/7**	Labor and delivery services are available 24 hours per day during the week and during weekends
Transportation	
**Motorized vehicle with motor fuel available for emergency referrals**	Facility has a functioning vehicle with fuel that is routinely available that can be used for emergency transportation or access to a vehicle in near proximity that can be used

Abbreviation: D&C, dilation and curettage.
